# The Effects of a Web-Based Intervention to Reduce Alcohol Consumption Among Middle-Aged Women: Protocol for a Randomized Controlled Trial

**DOI:** 10.2196/34842

**Published:** 2023-02-02

**Authors:** Mia Miller, Cassandra Wright, Emmanuel Kuntsche, Sandra Kuntsche

**Affiliations:** 1 Menzies School of Health Research Charles Darwin University Darwin Australia; 2 Centre for Alcohol Policy Research La Trobe University Melbourne Australia; 3 Burnet Institute Melbourne Australia

**Keywords:** alcohol consumption, web-based, online intervention, middle-aged women, Australia, intervention, alcohol research, alcohol, ecological momentary assessment, EMA

## Abstract

**Background:**

In the last decade, alcohol consumption among middle-aged women (40-65 years old) in Australia increased, despite declines in overall population consumption. Web-based, brief interventions are promising for reducing alcohol consumption, with efficacy shown in a wide range of populations. However, no published interventions have been designed specifically for and tested with middle-aged women.

**Objective:**

This study aims to design and implement a web-based intervention intended to reduce alcohol consumption among middle-aged women.

**Methods:**

The study is a 3-arm randomized controlled trial with a web-based intervention plus ecological momentary assessment (EMA) group compared to an EMA-only and a pre-post only control group. The study is aimed at middle-aged women, defined as women aged between 40 and 65 years, who consume alcohol at least weekly or who have consumed 4 or more drinks on 1 occasion in the last month. The intervention aims to reduce alcohol consumption through 4 modules that provide information on the health impacts of alcohol, mindfulness, social influences, and alcohol marketing. Intervention participants will also fill out biweekly EMA assessments. The comparators are EMA-only and pre-post control only. The primary outcome is alcohol consumption at 8 weeks compared between groups. Secondary outcomes are awareness of alcohol-related harms, readiness to change alcohol consumption, health status, mental health, and social support.

**Results:**

Ethics approval for this project was received on September 11, 2019. The trial was registered on August 14, 2020. Recruitment has commenced, and the expected results will be available in 2022.

**Conclusions:**

This web-based intervention aims to reduce alcohol consumption among middle-aged women, a currently understudied cohort in alcohol research.

**Trial Registration:**

Australia New Zealand Clinical Trials Registry (ANZCTR) ACTRN12620000814976; https://www.anzctr.org.au/Trial/Registration/TrialReview.aspx?ACTRN=12620000814976

**International Registered Report Identifier (IRRID):**

DERR1-10.2196/34842

## Introduction

### Background

Historically, men have been larger consumers of alcohol than women, but recent evidence has shown a gender convergence in rates of alcohol consumption [1]. In Australia, this is particularly notable among middle-aged women; rates of long-term risky drinking (measured as 2 or more standard drinks a day) have increased significantly among women aged 40-65 years from 8.8% in 2001 to 11.7% in 2019 [2]. Similarly, rates of risky single-occasion drinking (measured as 5 or more standard drinks on 1 occasion) rose from 13.5% to 19.8% over this same period [2]. This increase is of concern as women are more susceptible to the long-term negative health effects of alcohol including cardiovascular disease, cancer, and diabetes [3]. Women are also more likely to develop alcohol-use disorders from lower levels of consumption than men and are more vulnerable to the neurotoxic effects of alcohol [3].

Several systematic reviews have been carried out to determine the overall efficacy of web-based interventions aimed at reducing alcohol consumption. The most recent, a review of systematic reviews [4], found that web-based interventions were effective in reducing alcohol consumption, albeit effect sizes were mostly small, reflecting a weekly reduction of between 2 and 3 UK units (1 unit=8 grams of ethanol) or between 1 and 2.5 European units (1 unit=10 grams). Studies of interventions that have included women in the sample have often found that higher proportions of women than men take up the intervention [5], suggesting web-based interventions are an appropriate medium for female drinkers. Of studies demonstrating the efficacy of web-based interventions with women, the majority have only included college students [6,7].

There is a specific need for interventions designed by, and targeted at, middle-aged women for several reasons. Firstly, men and women have different patterns of alcohol use and different motives for consumption [3,8,9]. For example, women are lighter drinkers than men, and use alcohol to experience respite from their traditional female domestic and caring responsibilities. Further, most web-based interventions that are female-centric have been aimed at pregnant women or those of childbearing age, with the content heavily tailored to these specific groups and a particular focus on the harms of alcohol to unborn babies [10,11]. Lastly, it has been shown that tailoring interventions using co-design with a target population is particularly efficacious in ensuring the effectiveness of an intervention or program [12,13]; thus, an intervention designed by and for middle-aged women is likely to have amplified effectiveness.

To our knowledge, there are no web-based interventions targeted specifically at middle-aged women that have been documented in the literature. Our study aims to fill this gap by developing and testing the effectiveness of a web-based intervention in reducing alcohol consumption among women aged 40-65 years. The primary aim of this study is to test the effectiveness of a 3-arm web-based intervention in reducing both the frequency of alcohol consumption as well as the alcohol intake per occasion among middle-aged women. Secondary aims include testing the effectiveness of the intervention in increasing awareness of the long- and short-term harms associated with alcohol consumption, increasing motivation to reduce alcohol consumption, reducing alcohol-related harm, improving reported health status and mental health, and increasing social support. We will also test reactivity to the ecological momentary assessment (EMA) by comparing a control group with weekly assessment to a pre-post control group.

### Hypothesis 1

We hypothesize that by the end of the intervention period, participants in the web-based intervention group will report significantly lower alcohol consumption compared with participants in the pre-post control group (H1).

### Hypothesis 2

We hypothesize that by the end of the intervention period, participants in the web-based intervention group will display significant increases in their awareness of harms associated with alcohol consumption (H2a), their motivation to reduce their alcohol consumption (H2b), social support (H2c), and current health status (H2d); they will also display significant decreases in alcohol-related harm (H2e) when compared to the pre-post control only group.

### Hypothesis 3

We hypothesize that by the end of the intervention period, participants in the control group with weekly assessment will have greater reductions in alcohol consumption compared with participants in the pre-post control group (H3a). We further hypothesize that participants in the intervention group will report a greater reduction in alcohol consumption compared with participants in the control group with weekly assessment.

## Methods

### Study Design

This study is a 3-arm randomized controlled trial including 1 intervention group, 1 pre-post control group and 1 control group with weekly assessment. The trial was registered with Australia New Zealand Clinical Trials Registry (ACTRN12620000814976) on August 14, 2020.

### Ethics Approval and Consent to Participate

This study has received ethics approval from the La Trobe University Human Research Ethics Committee (HEC19938). Participants who are eligible for the intervention will be emailed a consent form with brief information about the study, prior to completing the baseline survey. Upon randomization, participants will complete a second short written consent form which will contain information specific to their intervention arm.

### Participants

For this study, participants will be eligible if they are aged between 40 and 65 years, identify as female, reside in the Australian Capital Territory or a surrounding postcode that covers the Australian Capital Territory, are able to understand written English, are able to provide informed consent, own a mobile phone with SMS text messaging capabilities, have access to the internet, and consume alcohol at least weekly or consume 4 or more standard drinks at least once a month.

Women will be ineligible for the study if they are currently receiving treatment for a substance use disorder or are not in Australia for the duration of the study.

### Sample Size Calculation

The effect sizes obtained in brief interventions, that is, change in the intervention group compared to the control group, are usually relatively small [4]. Power analyses in the statistical software G*Power 3.1.9.4 [14] were conducted to determine the power (probability) to successfully find differences between the intervention group and the 2 control groups. In this study, we aim to recruit 3000 participants. However, we may be short on this aim by one-third and have one-third attrition leaving us with a sample of 1333. Using repeated measures ANOVA with within-between interactions (ie, 2 measurements over time [pre-post assessments; note that in any case the EMA assessments will have a higher test power] interacting with the membership in 1 of 3 groups), this reduced sample will still have a power (probability) of more than 99% to detect even small effect sizes (*P*=.99; [15]) with an α-error threshold of .001. In other words, the intended sample size is sufficient to test all hypotheses successfully.

### Recruitment

Recruitment will be carried out through a multipronged approach using both “online” and “offline” access points. Online recruitment will occur through media outlets such as Facebook, Twitter, websites, web-based newsletters, and magazines. Offline recruitment will include advertisements in school newsletters, local community group newsletters, general practitioner clinics, libraries, and gyms. Advertisements will include a link and QR code for a brief web-based screening questionnaire to assess participant eligibility.

We will undertake rolling recruitment, such that participants will be recruited continuously until 3000 individuals are participating in the study. Each participant will undertake the baseline assessment immediately after recruitment to ensure retention.

### Procedures

This study is a 3-arm randomized controlled trial of a web-based intervention to reduce alcohol consumption in middle-aged women.

Potential participants responding to an advertisement will be linked to the study website, which will provide them with a brief description of the study and an eligibility screener. Participants who are eligible will be electronically sent an initial consent form with brief information about the study and will be asked to complete the baseline survey. Participants who do not respond to the initial prompt to complete the baseline survey will be sent 3 reminders over 2 weeks. Upon completion of the baseline survey, participants will be randomized into 1 of 3 groups through the back-end platform specifically designed for the intervention, using a computer-generated number allocated to each participant. Participants will then complete a second short consent form that contains information specific to their intervention arm. As with most behavioral interventions, although it is not possible to blind participants to the procedures relating to their own group, they will not be aware of the procedures relating to the other groups. The researchers responsible for analysis will also be blinded. Only 1 member of the research team, who will be the contact point for any participants experiencing technical difficulties or presenting with queries, will be aware of participant allocation, if required to provide support.

**Figure 1:**
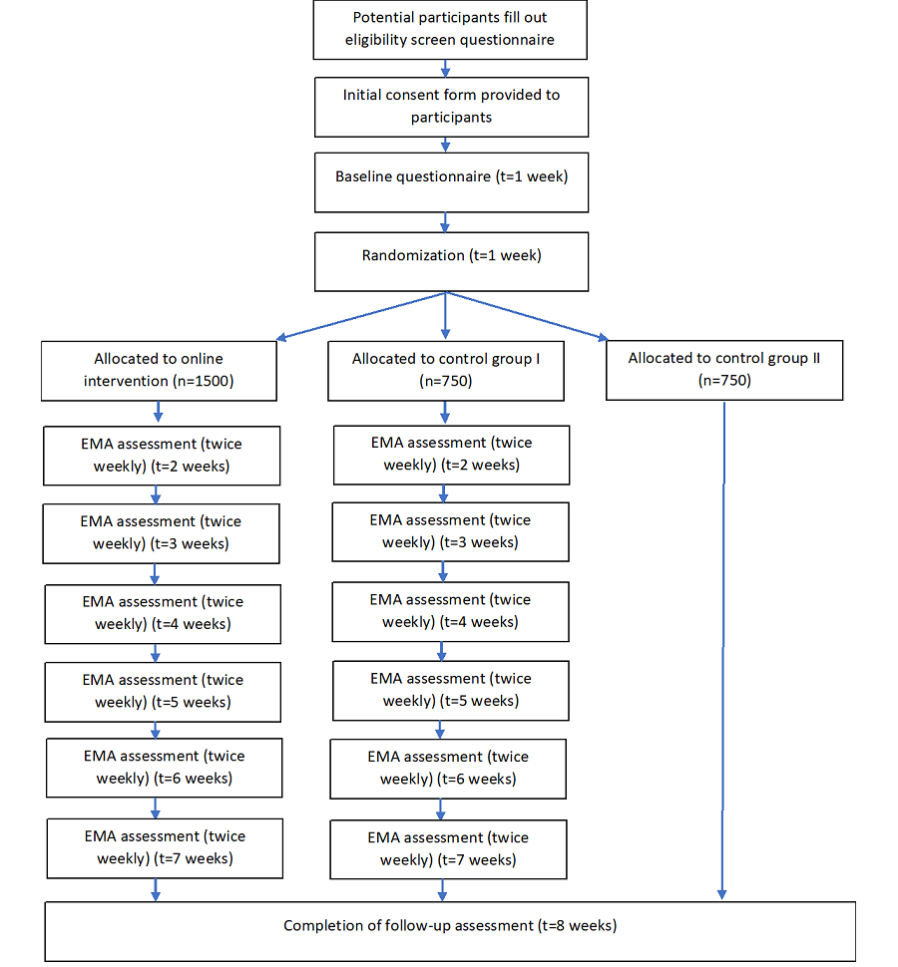
Intervention design. EMA: ecological momentary assessment; t: time point.

The study will include 3 parallel conditions, of which all 3 will complete a baseline and follow-up questionnaire. The intervention design is shown in Figure 1. Beyond that, the 3 conditions will include the experimental condition (web-based intervention and EMA), and 2 control groups (control group 1: baseline and follow-up assessment only, and control group 2: baseline and follow-up assessment plus EMA). They will undertake assessments of their alcohol consumption over an 8-week period, providing EMA measurements of their alcohol consumption on a twice-weekly basis (measuring their daily alcohol consumption retrospectively). Screenshots of the intervention are shown in Multimedia Appendix 1.

Participants will be able to withdraw from the intervention at their request. If a participant reports an adverse event or complaint, they will be unblinded by matching their participant ID with the contact details database to enable us to establish contact and respond to any issues accordingly. A risk management database will be maintained where records of any issues will be logged.

To promote retention, all participants who complete the study (ie, complete the final assessment measures) will be entered into a draw to win 1 of 24 vouchers. There will be 12 AUD $50 vouchers, 9 AUD $100 vouchers, and 3 AUD $250 vouchers available (AUD $1=US $0.67). The large number of vouchers available reflects the repeated engagement required of participants as well as the intended large number of participants.

### Intervention

The underlying theory for this web-based intervention is based on the assumptions of the Integrated Behavioral Model. This model posits that the most important determinant of behavior is the intention to perform the behavior, informed by individuals’ attitudes, perceived norms, and personal agency [16]. Within this model, intention to perform a behavior is modified by four key factors: knowledge and skills, environmental constraints, the salience of the behavior to the individual, and the role of habits [16]. The Transtheoretical Model of Change [17] will also be used to determine each participant’s readiness to change their alcohol use to understand how the intervention impacts motivation to change*.* The intervention also follows the principles of brief interventions for substance use [18]. Additionally, the intervention is based on the outcomes of interactive focus group workshops that were run by the research team with women aged 40-65 years.

### The Integrated Behavioral Model

Initially, participants will be surveyed on their alcohol use and provided with feedback on their current consumption, in line with brief intervention practice. Following this, participants will *set goals* around their intentions to reduce their alcohol use over the period of the intervention in order to promote personal agency or self-efficacy. Participants will also be able to *track their alcohol use* throughout the intervention period to become more aware of their habits and behaviors. Participants will work through four *modules of content*, which will provide information aimed at changing participants’ attitudes and perceived norms. This will include the following: (1) the Low-Risk Drinking module, which will provide evidence on the harms of alcohol use and information on what a standard drink is; (2) the Mindful Drinking module, which will provide information about drinking motives and provide participants with skills to both learn to consume alcohol mindfully and to apply the broader techniques of mindfulness to their lives to reduce the common antecedents of alcohol use (such as stress); (3) the Social Drinker module, which will explore the social influences of drinking, including facts about how people influence each other both consciously and unconsciously, encourage participants to reflect on where alcohol currently fits into their social lives, and help build participant’s skills to talk about their alcohol consumption with others; and (4) the Alcohol is Everywhere module, which will look at the broader factors that influence alcohol consumption, including information about the normativity of alcohol in Australian society and how alcohol availability and outlet density impact consumption, the ways in which the alcohol industry specifically targets women, how alcohol advertising impacts children, and the broader alcohol industry tactics used to promote their products. The overall aim of the content is to make participants more aware of their alcohol use and the impacts it has on their lives and on the lives of others, in order to produce attitudinal and normative changes, while building skills to cope with stress and increase self-efficacy for reducing consumption in social situations.

To improve participants’ knowledge and skills to reduce their alcohol use, and to remove environmental constraints, each module will have between 1 and 3 associated *challenges*. The challenges include, for example, an opportunity for participants to write a script for a conversation with a friend or family member on how to say no when offered alcohol, information on alternate activities participants can do to relax, and an opportunity to track how they engage in these activities over a 1-week period.

Participants will be sent reminders to complete their assessments, and upon completion of the assessment, will be redirected back to the intervention content. Participants can complete the intervention content in any order and will have the entire intervention period to access and work through all of the content.

At the conclusion of the trial, participant summaries will be developed and distributed to those participants who request one.

### Comparator

Participants in control group 1 will not be contacted again until the 8-week follow-up questionnaire. Participants in control group 2 (baseline and follow-up assessment plus EMA) will undertake the web-based repeated measures assessment of their alcohol consumption retrospectively twice a week. After they are recruited and randomized, these participants will be sent a link via text message to a web-based EMA. The assessment on Sunday at 12 PM will ask them about their drinking patterns over the weekend (ie, Thursday afternoon until Sunday morning), and the assessment on Thursday at 12 PM will ask about their drinking patterns during the week (ie, Sunday afternoon until Thursday morning). All participants in the control groups will be given access to the web-based intervention at the completion of the study.

### Outcomes

The primary outcomes include frequency of alcohol consumption, as well as alcohol intake per drinking occasion. The secondary outcomes are awareness of alcohol-related harms, willingness to change alcohol consumption, alcohol-related harm, current health status, mental health, and social support or loneliness.

### Measures

The following measures will be used, administered at baseline, EMA, and follow-up.

#### Baseline Survey: Demographic Questions

The baseline survey will assess a series of demographic characteristics, including educational level, country of birth, marital status, income, whether the participating women have children, the number of dependent children living in the household, employment status, as well as smoking status and frequency.

#### Alcohol-Related Measures

##### Typical Drinking (Baseline and Follow-up)

Participants will also be asked to report on how much they typically drink each day of the week (eg, “How many drinks containing alcohol do you consume on a typical Monday?”)

##### Frequency of Risky Single-Occasion Drinking (Baseline and Follow-up)

Frequency of binge drinking will be assessed by asking participants how often they have had 4 or more standard drinks (containing 10 grams of pure ethanol) on 1 drinking occasion over the last 12 months.

##### Daily Alcohol Consumption (Baseline, EMA, and Follow-up)

Participants’ daily alcohol consumption will be measured using a drinking diary, where twice a week they will retrospectively report on the number of beverages they consumed each day (eg, “Thinking back over the last three days, how many standard drinks containing alcohol did you have on each day of the week?”)

##### Alcohol-Related Harm (Baseline and Follow-up)

A total of 15 items from the Rutgers Alcohol Problem Index [19] and the Young Adult Alcohol Consequences Questionnaire [20] were selected based on their age-appropriateness to our target group. Participants will be asked the following: “How many times has this happened to you while you were drinking or because of your drinking during the last year?”: “Got into fights with other people,” “Went to work drunk,” “Missed out on other things because you spent too much money on alcohol,” “Caused shame or embarrassment to someone,” “Neglected your responsibilities,” “Friends or relatives avoided you,” “Felt that you needed more alcohol than you used to in order to get the same effect,” “Tried to control your drinking (tried to drink only at certain times of the day or in certain places, that is, tried to change your pattern of drinking),” “Had withdrawal symptoms, that is, felt sick because you stopped or cut down on drinking,” “Noticed a change in your personality,” “Felt that you had a problem with alcohol,” “Missed a day (or part of a day) of work,” and “Kept drinking when you promised yourself not to,” with four possible answer categories ranging from 0 (none) to 3 (more than 5 times).

##### Pressures From Different Sources to Drink More and to Drink Less (Baseline and Follow-up)

Two questions on pressures to drink have been adapted from the core questionnaire from the GenACIS project [21], which are as follows: “During the last 12 months, have any of the following persons attempted to influence your drinking so that you would drink less or cut down?” and “During the last 12 months, have you felt influenced to drink or drink more by someone who drinks more than you do?” with each question having the following response options: “Your spouse/partner/romantic partner?” “Your child or children?” “Some other female member of your family?” “Some other male member of your family?” “Someone at your work?” “A female friend or acquaintance?” “A male friend or acquaintance?” and “A doctor or health worker?” with yes or no answer categories for each option.

##### Awareness of Alcohol Harms (Baseline and Follow-up)

As no validated scale currently exists to measure awareness of alcohol harms, 2 questions will be taken from the scale used in the 2017 study by Coomber et al [22]: (1) “Which of the following do you think are likely consequences of a single occasion of heavy drinking (ie, short-term consequences)?” with participants asked to rank the accuracy (ranging from definitely untrue to definitely true) of the following consequences: “Lack of coordination and slower reflexes,” “Reduced concentration,” “Motor vehicle, bicycle and pedestrian accidents,” “Injuries associated with falls, accidents, violence and intentional self-harm,” “Alcohol poisoning,” “Drownings,” “Coma and death,” and (2) “Which of the following do you think are likely consequences of consuming alcohol over many years (ie, long-term consequences)?” with participants asked to rank the accuracy of the following consequences: “Harm to unborn babies,” “Cirrhosis of the liver,” “Brain damage,” “Stomach problems,” “Heart and blood disease,” “Pancreatitis,” “Bowel cancer,” “Pharyngeal cancer,” “Oesophageal cancer,” “Mouth cancer,” “Larynx cancer,” and “Breast cancer.”

##### Drinking Motives Questionnaire (Baseline and Follow-up)

Drinking motives will be assessed using the Drinking Motives Questionnaire-Revised [23].

#### Baseline and Follow-up Survey: Other Measures

##### Readiness to Change (Baseline and Follow-up)

Readiness to change will be measured using the 12-item Readiness to Change Questionnaire [24].

##### Current Level of Stress (Baseline, EMA, and Follow-up)

Current level of stress will be measured using the 4-item Perceived Stress Scale [25].

##### Self-efficacy (Baseline, EMA, and Follow-up)

Self-efficacy will be measured using the 10-item General Self-Efficacy Scale [26].

##### Relationship Quality (Baseline and Follow-up)

Relationship quality will be measured using the second item of the Relationship Assessment Scale [27], “In general, how satisfied are you with your relationship?” with five possible answer categories ranging from 1 (not satisfied) to 5 (very satisfied).

##### Current Health Status (Baseline and Follow-up)

Current health status will be measured using the first item of the General Health Questionnaire [28]— “In general, would you say your health is,” with five possible answer categories ranging from 1 (poor) to 5 (excellent).

##### Mental Health (Baseline and Follow-up)

Mental health will be measured using the 5-item Mental Health Inventory [29].

##### Social Support or Loneliness (Baseline, EMA, and Follow-up)

Social support or loneliness will be measured using the 8-item UCLA Loneliness Scale-8 [30].

##### Current Use of Technology (Baseline)

Five items from the Media and Technology Usage and Attitudes Scale [31] will be used to capture attitudes toward technology. Participants will be asked how strongly they agree with the following questions: “I feel it is important to be able to access the Internet anytime I want,” “I think it is important to keep up with the latest trends in technology,” “I get anxious when I don’t have my cell phone,” “I get anxious when I don’t have the internet available to me,” and “New technology makes people waste too much time.” Participants will also be asked to report how many hours per week on average they spend on their computer (for leisure and for work), on their mobile phone, on a tablet, searching the internet on any device, and using apps on any device.

##### Client Satisfaction Survey (Follow-up)

The 8-item Client Satisfaction Questionnaire Adapted for Online Interventions [32] will be used.

##### Participant’s Use of Intervention (Follow-up)

The intervention’s back-end system will record the number of times participants log in to view the intervention content, and the time they spend on the website each time they log in. It will also record whether participants complete the requested number of follow-up EMAs. These data (number of visits, time spent on website, and number of EMAs completed) will be extracted from the system to assist in assessing the fidelity of the implementation of the intervention.

### Data Management

Participants will complete all assessments and access the intervention online; data will be stored on a secure server based in Australia. Access to the data will be password protected and limited to the members of the research team.

### Data Analysis

The reporting and presentation of this trial will be in accordance with the CONSORT (Consolidated Standards of Reporting Trials) guidelines for randomized controlled trials, and data on screening, refusals, and dropout will be coded and reported on per the guidelines [33]. The comparative analysis will be analyzed in accordance with the intention-to-treat principle and the completers-only framework. Participants will have to sign up to the intervention and complete approximately 30% of the EMA surveys to be considered for inclusion in the analysis; missing data will be handled accordingly. The intention-to-treat analyses’ multiple imputation methods will be used, while completers-only analyses will be conducted on participants providing scores for all measurements.

All data that are collected will be analyzed using statistical software packages such as STATA (StataCorp) and Mplus (Muthén & Muthén). ANOVAs, contrasting the intervention groups against both control groups, will be run, and logistic and linear regression models will be used to determine potential influencing variables. Longitudinal analyses will be conducted to test group differences in EMA assessments over time.

Besides testing the main effects of the intervention, moderating effects of age, drinking status (whether participants are “risky” drinkers according to the Australian Alcohol Guidelines [34]), educational status, and presence of dependent children in the household will be investigated to establish whether specific subgroups are more likely to benefit from the intervention.

## Results

Ethics approval for this project was received on September 11, 2019. The trial was registered on August 14, 2020. Recruitment has commenced, and the expected results will be available in 2022.

## Discussion

### Expected Outcomes

This paper describes the study protocol of a randomized controlled trial to assess the benefit of a targeted web-based intervention to reduce alcohol consumption among middle-aged women. Intervention with this group is imperative because their alcohol consumption has increased at a time when that of other groups has remained stable or declined [35]. The primary aim of the study is to test whether a web-based intervention can reduce alcohol consumption, increase awareness of alcohol-related harms, increase motivation to reduce alcohol consumption, increase social support and reported health status, and decrease alcohol-related harms, when compared to a no-intervention control group.

### Strengths and Limitations

Most web-based interventions aimed at reducing alcohol consumption have been designed for the general adult population, youth, or pregnant women. This study will be, to our knowledge, the first web-based intervention designed by and tested on middle-aged women. Additionally, the use of repeated-measured assessment (EMA) will allow us to gain rich data on participants’ alcohol consumption over an 8-week period, which is a strength. Further, the use of 2 control groups (1 with EMA and 1 without) will enable us to assess the true effect of the intervention, as well as determine whether EMAs alone may result in reduced alcohol consumption. As with most alcohol research, the reliance on self-report data can lead to reporting bias; however, it is not anticipated that this bias will differ among the 3 groups. The intervention will only be tested initially on women residing in 1 Australian state, so generalizability may be limited in the first instance.

## Appendix

Multimedia Appendix 1 Screenshots of intervention.

## Abbreviations

CONSORT: Consolidated Standards of Reporting Trials
EMA: ecological momentary assessment

## Availability of Data and Materials

The data generated and analyzed during the study are not publicly available due to privacy issues but are available from the corresponding author on reasonable request.

## References

[1] Slade T, Chapman C, Swift W, Keyes K, Tonks Z, Teesson M (2016). Birth cohort trends in the global epidemiology of alcohol use and alcohol-related harms in men and women: systematic review and metaregression. BMJ Open.

[2] Miller M, Mojica-Perez Y, Livingston M, Kuntsche E, Wright CJC, Kuntsche S (2022). The who and what of women's drinking: Examining risky drinking and associated socio-demographic factors among women aged 40-65 years in Australia. Drug Alcohol Rev.

[3] Erol A, Karpyak VM (2015). Sex and gender-related differences in alcohol use and its consequences: Contemporary knowledge and future research considerations. Drug Alcohol Depend.

[4] Sundström C, Blankers M, Khadjesari Z (2017). Computer-Based Interventions for Problematic Alcohol Use: a Review of Systematic Reviews. Int J Behav Med.

[5] White A, Kavanagh D, Stallman H, Klein B, Kay-Lambkin F, Proudfoot J, Drennan J, Connor J, Baker A, Hines E, Young R (2010). Online alcohol interventions: a systematic review. J Med Internet Res.

[6] Kypri K, Mccambridge J, Vater T, Bowe S, Saunders J, Cunningham J, Horton N (2012). Web-based intervention for Maori university students with hazardous drinking: double-blind, multi-site randomised controlled trial. Inj Prev.

[7] Kypri K, Hallett J, Howat P, McManus A, Maycock B, Bowe S, Horton NJ (2009). Randomized controlled trial of proactive web-based alcohol screening and brief intervention for university students. Arch Intern Med.

[8] O'Brien H, Callinan S, Livingston M, Doyle JS, Dietze PM (2020). Population patterns in Alcohol Use Disorders Identification Test (AUDIT) scores in the Australian population; 2007-2016. Aust N Z J Public Health.

[9] Emslie C, Hunt K, Lyons A (2012). Older and wiser? Men's and women's accounts of drinking in early mid-life. Sociol Health Illn.

[10] van der Wulp NY, Hoving C, Eijmael K, Candel MJ, van Dalen W, De Vries H (2014). Reducing alcohol use during pregnancy via health counseling by midwives and internet-based computer-tailored feedback: a cluster randomized trial. J Med Internet Res.

[11] Nayak MB, Kaskutas LA, Mericle AA (2019). Randomized Trial of an Innovative Electronic Screening and Brief Intervention for Reducing Drinking Among Women of Childbearing Age. J Addict Med.

[12] Yardley L, Spring BJ, Riper H, Morrison LG, Crane DH, Curtis K, Merchant GC, Naughton F, Blandford A (2016). Understanding and Promoting Effective Engagement With Digital Behavior Change Interventions. Am J Prev Med.

[13] Slattery P, Saeri AK, Bragge P (2020). Research co-design in health: a rapid overview of reviews. Health Res Policy Syst.

[14] Faul F, Erdfelder E, Lang A, Buchner A (2007). G*Power 3: a flexible statistical power analysis program for the social, behavioral, and biomedical sciences. Behav Res Methods.

[15] Cohen J (1988). Statistical Power Analysis for the Behavioural Sciences.

[16] Fishbein M (2016). An integrative model for behavioral prediction and its application to health promotion. Emerging Theories in Health Promotion Practice and Research.

[17] Prochaska JO, DiClemente CC (1982). Transtheoretical therapy: Toward a more integrative model of change. Psychotherapy: Theory, Research & Practice.

[18] O'Donnell A, Anderson P, Newbury-Birch D, Schulte B, Schmidt C, Reimer J, Kaner E (2014). The impact of brief alcohol interventions in primary healthcare: a systematic review of reviews. Alcohol Alcohol.

[19] White HR, Labouvie EW (1989). Towards the assessment of adolescent problem drinking. J Stud Alcohol.

[20] Read JP, Kahler CW, Strong DR, Colder CR (2006). Development and preliminary validation of the young adult alcohol consequences questionnaire. J Stud Alcohol.

[21] Wilsnack S (2012). The GENACIS project: a review of findings and some implications for global needs in women-focused substance abuse prevention and intervention. SAR.

[22] Coomber K, Mayshak R, Curtis A, Miller PG (2017). Awareness and correlates of short-term and long-term consequences of alcohol use among Australian drinkers. Aust N Z J Public Health.

[23] Cooper ML (1994). Motivations for alcohol use among adolescents: Development and validation of a four-factor model. Psychological Assessment.

[24] Rollnick S, Heather N, Gold R, Hall W (1992). Readiness to Change Questionnaire: User's Manual. British Journal of Addiction.

[25] Cohen S, Kamarck T, Mermelstein R (1983). A Global Measure of Perceived Stress. Journal of Health and Social Behavior.

[26] Schwarzer R, Jerusalem M (1995). Generalised Self-Efficacy Scale. ResearchGate.

[27] Hendrick SS, Dicke A, Hendrick C (2016). The Relationship Assessment Scale. Journal of Social and Personal Relationships.

[28] Goldberg DP, Hillier VF (1979). A scaled version of the General Health Questionnaire. Psychol Med.

[29] Berwick DM, Murphy JM, Goldman PA, Ware JE, Barsky AJ, Weinstein MC (1991). Performance of a five-item mental health screening test. Med Care.

[30] Hays R, DiMatteo MR (1987). A short-form measure of loneliness. J Pers Assess.

[31] Rosen L, Whaling K, Carrier L, Cheever N, Rokkum J (2013). The Media and Technology Usage and Attitudes Scale: An empirical investigation. Comput Human Behav.

[32] Boß L, Lehr D, Reis D, Vis C, Riper H, Berking M, Ebert DD (2016). Reliability and Validity of Assessing User Satisfaction With Web-Based Health Interventions. J Med Internet Res.

[33] Schulz K, Altman D, Moher D, CONSORT Group (2010). CONSORT 2010 Statement: updated guidelines for reporting parallel group randomised trials. BMC Med.

[34] (2020). Australian Guidelines to Reduce Health Risks from Drinking Alcohol. National Health and Medical Research Council.

[35] Livingston M, Callinan S, Dietze P, Stanesby O, Kuntsche E (2018). Is there gender convergence in risky drinking when taking birth cohorts into account? Evidence from an Australian national survey 2001-13. Addiction.

