# Supplementary material for: The Effects of a Web-Based Intervention to Reduce Alcohol Consumption Among Middle-Aged Women: Protocol for a Randomized Controlled Trial
Source: JMIR Res Protoc. 2023 Feb 2;12:e34842. doi: 10.2196/34842 (PMC9936363; doi:10.2196/34842)
Supplement: Multimedia Appendix 1 [file resprot_v12i1e34842_app1.pdf]

Multimedia Appendix 1

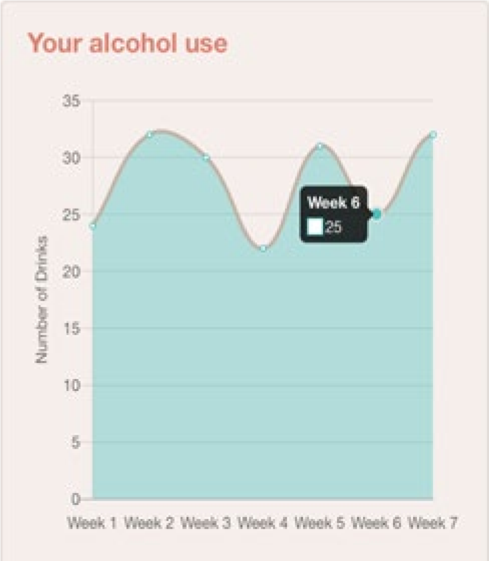

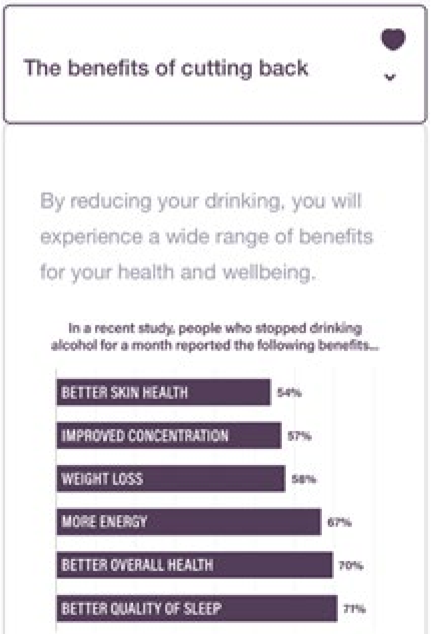

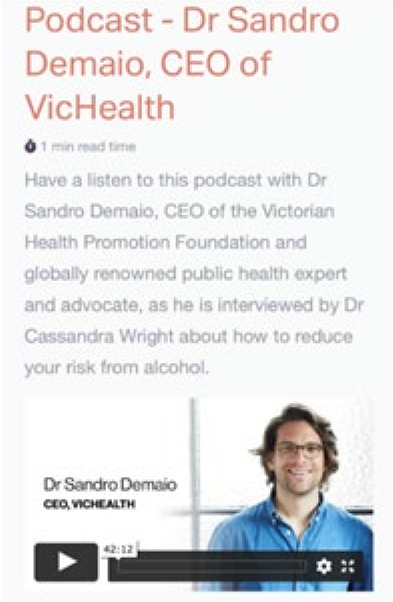

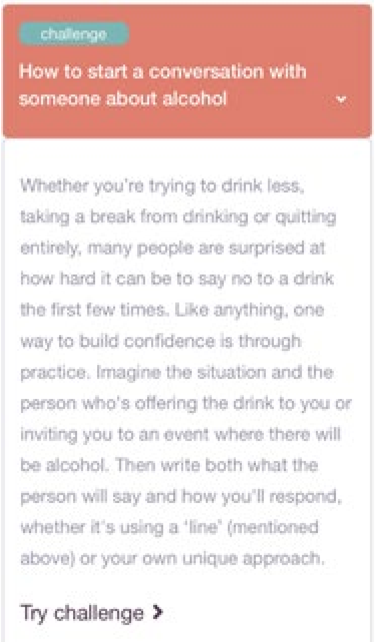

This is a Multimedia Appendix to a full manuscript published in the Journal of Medical Internet Research.
